# Postnatal Ethanol-Induced Neurodegeneration Involves CB1R-Mediated β-Catenin Degradation in Neonatal Mice

**DOI:** 10.3390/brainsci10050271

**Published:** 2020-05-01

**Authors:** Shivakumar Subbanna, Balapal S. Basavarajappa

**Affiliations:** 1 Division of Analytical Psychopharmacology, Nathan Kline Institute for Psychiatric Research, 140 Old Orangeburg Rd, Orangeburg, NY 10962, USA; Subbanna.Shivakumar@nki.rfmh.org; 2 New York State Psychiatric Institute, New York, NY 10032, USA; 3 Department of Psychiatry, College of Physicians & Surgeons, Columbia University, New York, NY 10032, USA; 4 Department of Psychiatry, New York University Langone Medical Center, New York, NY 10016, USA

**Keywords:** alcohol, development, apoptosis, FASD, Wnt signaling

## Abstract

Alcohol consumption by pregnant women may produce neurological abnormalities that affect cognitive processes in children and are together defined as fetal alcohol spectrum disorders (FASDs). However, the molecular underpinnings are still poorly defined. In our earlier studies, we found that ethanol exposure of postnatal day 7 (P7) mice significantly induced widespread neurodegeneration mediated via endocannabinoids (eCBs)/cannabinoid receptor type 1 (CB1R). In the current study, we examined changes in the β-catenin protein levels that are involved in the regulation of neuronal function including neuronal death and survival. We found that moderate- and high-dose postnatal ethanol exposure (PEE) significantly reduced active-β-catenin (ABC) (non-phosphorylated form) protein levels in the hippocampus (HP) and neocortex (NC). In addition, we found that moderate- and high-dose PEE significantly increased the phosphorylated-β-catenin (p-β-catenin)/ABC ratios in the HP and NC. Antagonism/null mutation of CB1R before PEE to inhibit CC3 production mitigated the loss of ABC protein levels. Collectively, these findings demonstrated that the CB1R/β-catenin signaling mechanism causes neurodegeneration in neonatal mouse brains following PEE.

## 1. Introduction

Alcohol exposure during pregnancy can cause abnormal fetal development and functional anomalies to multiple brain regions that result in a broad spectrum of neurobehavioral and cognitive deficits termed fetal alcohol spectrum disorders (FASD) [1,2]. The prevalence of FASDs in the United States and several Western European countries may be as high as 2–5% [3]. The rise in the frequency of FASD is a critical factor in the upsurge of children and adults with non-genetic intellectual disability in Western nations [4]. FASD is associated with reduced intellectual ability and behavioral abnormalities [5,6,7,8,9]. In rodents, a significant amount of third trimester-equivalent [10] brain growth occurs following birth [11,12], and rapid synaptic expansion occurs during postnatal days 4–10 (P4–10). Hence, in binge ethanol models, ethanol has been directly administered to neonatal pups to examine the effects of ethanol use during the third trimester of fetal development in humans [13]. Postnatal ethanol exposure (PEE) in postnatal day 7 mice (P7) causes extensive neurodegeneration (activation of caspase-3) in several brain regions including the hippocampus (HP) and neocortex (NC) [14], which are vital for learning and memory [15]. As ethanol lacks a specific receptor, the mechanism by which ethanol initiates apoptosis in the postnatal cell population is unknown. Most importantly, the PEE paradigm causes persistent synaptic, learning, and memory behavioral deficits in adulthood [16,17,18,19,20,21]. Although several mechanisms have been implicated in PEE-induced neurodegeneration in neonatal mice, our recent studies using pharmacological, genetic, and epigenetic tools suggest a significant role for the endocannabinoid (eCB)/cannabinoid receptor type 1 (CB1R) signaling pathways [22].

The eCB signaling system, containing endocannabinoids, cannabinoid receptors type 1 and 2, and the enzymes involved in their metabolism [23,24], is a ubiquitous signaling system involved in the regulation of cell fate [25,26]. The eCB system regulates synaptic events in developing [19,20,27,28] and adult brains [24]. eCBs and CB1R were strongly suggested to have crucial functions in neuronal maturation during brain development [29,30]. PEE activates several components of the eCB system, leading to neurodegeneration in neonatal mice and persistent synaptic, learning, and memory abnormalities in adult mice [19,20,31,32]. In addition to enhanced eCB such as anandamide (AEA) and related biosynthetic enzymes, the PEE-induced transcriptional activation of the Cnr1 gene, a gene encodes CB1R protein, followed by enhanced Cnr1 mRNA and CB1R protein expression in cortical and hippocampal regions. The administration of SR141716A (SR) to block CB1R or the genetic deletion of CB1R (CB1R KO) before PEE rescued neuronal apoptosis. Remarkably, synaptic plasticity, learning, and memory are impaired by PEE and are later restored by SR treatment or genetic deletion of CB1R. The enhanced AEA/CB1R signaling pathway may be directly linked to the neurobehavioral abnormalities found in FASD [For recent review see [33]]. Activation of CB1R has been shown to inhibit the Wnt/β-catenin signaling pathway [34]. The binding of Wnt ligands to Frizzled (Fz) family receptors and low-density lipoprotein receptor (LDLR)-related protein 5 (LRP5) and (LDLR)-related protein 6 (LRP6) leads to activation of the canonical Wnt pathway via stabilization of β-catenin in the cytoplasm. Stabilized β-catenin translocates to the nucleus [35,36,37]. In the nucleus, β-catenin interacts with T-cell factor (TCF)/Lef transcription factors and leads to the transcription of target genes that promote cell proliferation and differentiation [38]. In the absence of Wnt ligands, β-catenin undergoes phosphorylation by a multiprotein degradation complex, making β-catenin susceptible to ubiquitination and degradation by the proteasome. Furthermore, β-catenin signaling has been shown to function in development, cell proliferation, and cell survival [39,40] and has been implicated in several psychiatric and neurodegenerative disorders [41,42]. In this study, we examined whether PEE, which activates several components of the eCB system leading to neurodegeneration in neonatal mice, also inhibits the β-catenin signaling pathway in P7 mice. The findings suggest that PEE destabilizes β-catenin through enhanced phosphorylation of β-catenin followed by degradation, and this change is rescued by inhibition of CB1R.

## 2. Materials and Methods

### 2.1. Animals

Male and female C57BL/6J and CB1R heterozygous mice were housed in typical (12 h light/12 h dark cycle) laboratory conditions. Mice were allowed to ad libitum access to food and water. CB1R heterozygous mice were backcrossed on the C57BL/6J background for over 10 generations. We generated CB1R wild-type (WT) and knockout (KO) mice from a described CB1R heterozygous (created by Dr. Andreas Zimmer from the NIMH, lacking the functional CB1R gene in all tissues) [43] breeding colony at the Nathan Kline Institute. The CB1RWT and KO mouse genotypes were assessed as described previously [44]. All protocols were approved by the NKI Institutional Animal Care and Use Committee (# AP2018-616).

### 2.2. Ethanol and SR141716A (SR) Administration

In the present study, we used the PEE paradigm, in which acute ethanol treatment induces widespread neurodegeneration in many brain regions, including the hippocampus and cortex, without causing any lethality [45]. Half of the male and female 7-day-old (based on the day of birth) C57BL/6J, CB1R KO or CB1RWT mice from each litter were injected subcutaneously (s.c.) with saline and the other half with ethanol (1.0 g/kg (moderate-dose) or 2.5 g/kg (high-dose), s.c. at 0 h and again at 2 h) using a previously described method [20,46,47]. In some experiments, the C57BL/6J mice were preadministered an optimum dose (1 mg/kg) of the CB1R antagonist SR141617A to block CB1R activity. In our earlier studies, we demonstrated that preadministration of 1 mg/kg completely prevented the ethanol-induced activation of caspase-3 in P7 mice [20]. SR (gift from RBI, Natick, MA) was dissolved in 10 μL of ethanol followed by 10 μL of Tween 80, and then, the volume was made up with a sterile saline solution. The SR solution was administered (1 mg/kg) by s.c. administration at a volume of 5 μL/g body weight 30 min before ethanol administration. The vehicle solution was injected as an SR control. The blood ethanol levels (BEL) in all treated P7 mouse sera were monitored using a standard alcohol dehydrogenase-based method [48]. In kinetic studies, saline was injected instead of ethanol for 0 h of treatment. Each experiment used 6–8 pups/group. Animals were killed by decapitation, and hippocampus (HP) and neocortex (NC) samples were dissected, flash-frozen, stored at −80 °C and used for all the studies.

### 2.3. Immunohistochemistry (IHC)

Mice were perfused with a solution containing 4% paraformaldehyde and 4% sucrose in 0.05 M cacodylate buffer (pH 7.2) at 8 h after the first dose of saline or ethanol administration. This time point was shown to exhibit maximum caspase-3 activation (in one or more brain regions) in previous studies [45,46,47]. The free-floating sections were processed according to our previously described protocols [45,46,47] and immunostained with an antibody against cleaved caspase-3 (Asp175) (CC3) (Cell Signaling Technology, Danvers, MA, USA) and ABC reagents (Vectastain ABC Elite Kit, Vector Labs, Burlingame, CA, USA) and a peroxidase substrate (DAB) kit (Vector Labs) were used to label neurodegenerating neurons. For secondary Ab specificity, the primary antibodies were omitted from the reactions. Also, pre-incubation with blocking peptides for the anti-CC3 (GenScript, Piscataway, NJ, USA) completely blocked the immunostaining of the CC3 antibody. All photomicrographs were captured using a 2.5 ×, or 40 × objective with a Nikon Eclipse TE2000 inverted microscope with a digital camera (DXM1200F, Morrell Instrument Company, Melville, NY, USA).

### 2.4. Western Blotting Analysis

At 4 to 24 h after the first saline or ethanol injection, the HP and NC tissues were subjected to homogenization using buffer (0.1 M Tris, 1.25 mM sucrose, 25 mM KCl, 0.5 mM PMSF, 0.1 M sodium fluoride, 0.1 M β-glycerol phosphate, 25 mM NaVO4, pH 7.5) containing freshly added 1% protease inhibitor mixture (Roche, Indianapolis, IN, USA). The HP and NC homogenates were handled as described previously [20,49]. The HP and NC tissue homogenates were centrifuged at 7700× *g* for 1 min, and the supernatant was aspirated and stored at −80 °C until use. The nuclear pellet was then resuspended in a nuclear extraction reagent (NER) (# 78833, Thermo Fisher Scientific, Suwanee, GA, USA) [50]. The nuclear fraction was prepared [according to the manufacturer’s instructions (Thermo Fisher Scientific, Waltham, MA, USA)] by suspending the nuclear pellet in ice-cold NER, and the samples were vortexed for 15 s. Then, the samples were placed on ice and vortexed for 15 s every 10 min for a total of 40 min. The samples were sonicated for 30 s followed by centrifugation at 16,000× *g* for 10 min (4 °C). The supernatant was collected in prechilled tubes and stored at −80 °C for further studies. The samples were prepared in a sample buffer as previously described by our laboratory [20,51]. In all immunoblot experiments, blots were stained with Ponceau S to confirm equal loading in each lane before further processing. Blots were incubated at room temperature for 3 h or at 4 °C overnight with the following individual primary antibodies: anti-mouse-active-β-catenin (05-665; anti-ABC, clone 8E7; 1:1000) (EMD Millipore, Billerica, MA, USA), anti-rabbit-p-β-catenin (monoclonal; Ser33/37/Thr41; #9561, 1:1000) and anti-mouse-β-actin (#3700, 1:5000, Cell Signaling Technology) and processed as previously described by our laboratory [20,51]. The β-catenin antibodies specificity was determined by pre-incubating β-catenin antibody with an excess amount of β-catenin peptide (#1002, #1120, Cell Signaling Technology). Blots were incubated with a secondary antibody (goat anti-mouse peroxidase conjugate, #AP 124P, 1:5000; goat anti-rabbit, #AP132P, 1:5000, EMD Millipore) alone as a control and produced no bands.

## 3. Statistical Analysis

The experiments were performed using an equal number of animals per treatment. All the data are shown as the mean ± SEM. A statistical analysis of the data was performed by either a one-way analysis of variance ANOVA or a two-way ANOVA with Bonferroni’s *post hoc* test. A *p* < 0.05 cutoff was used to represent statistical significance in all the comparisons. Prism software (GraphPad, San Diego, CA, USA) was used to perform the statistical analyses.

## 4. Results

**Figure 1:**
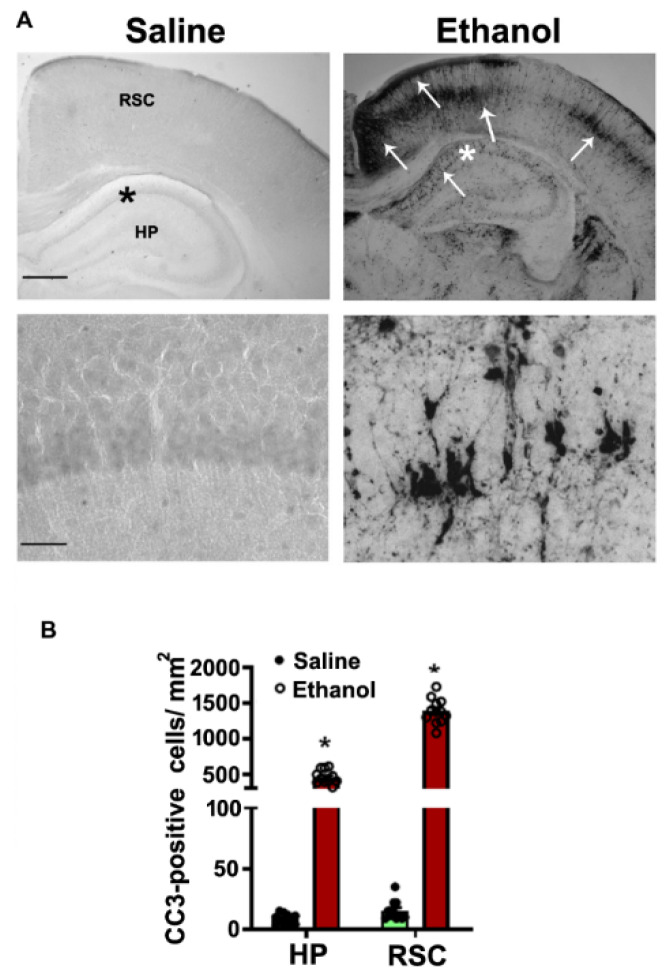
Enhanced CC3-positive cells in the P7 mouse HPand NC brain regions in response to high-dose ethanol exposure. The free-floating coronal brain sections (HP, and RSC (retrosplenial cortex)) were obtained after saline and 8 h ethanol-exposed mice and sections were subjected to IHC analysis with anti-rabbit-CC3 (**A**). The arrows indicate the CC3-positive neurons in the HP and RSC. Scale bars = 200 μm. The hippocampal region was enlarged to show the CC3-positive cells (*). CC3-positive cells were counted in the HP and RSC brain regions (**B**). Error bars, SEM (* *p* < 0.05 vs. the saline group, *n* = 6 pups/group).

The P7 mice were administered a moderate (1.0 g/kg, s.c.) or high (2.5 g/kg, s.c.) dose of ethanol at 0 h and again at 2 h. The BELs were determined at 3 and 9 h after first dose ethanol treatment. Consistent with an earlier finding [46], we observed BELs of 0.21 ± 0.023 g/dl at 3 h that were steadily reduced to 0.089 ± 0.012 g/dl at 9 h after the first moderate -dose ethanol administration. Moreover, similar to previous findings [20,47], our observations showed BELs of 0.44 ± 0.02 g/dl at 3 h that were steadily reduced to 0.26 ± 0.01 g/dl at 9 h after the first high-dose ethanol administration. We also performed cleaved caspase-3 immunostaining (generation of CC3 as a marker for neurodegeneration) in the brains of the P7 mice 8 h after the first moderate or high dose of ethanol or saline administration. Both moderate- [46] and high-dose ethanol [14,20,47,52,53] exposure paradigms recapitulated earlier findings, and moderate-dose ethanol administration induced mild caspase-3 activation (data not shown), whereas high-dose ethanol triggered robust, extensive caspase 3 activation (Figure 1).

### 4.1. P7 Ethanol Exposure Reduces the Cytosolic ABC Levels in the HP and NC

**Figure 2:**
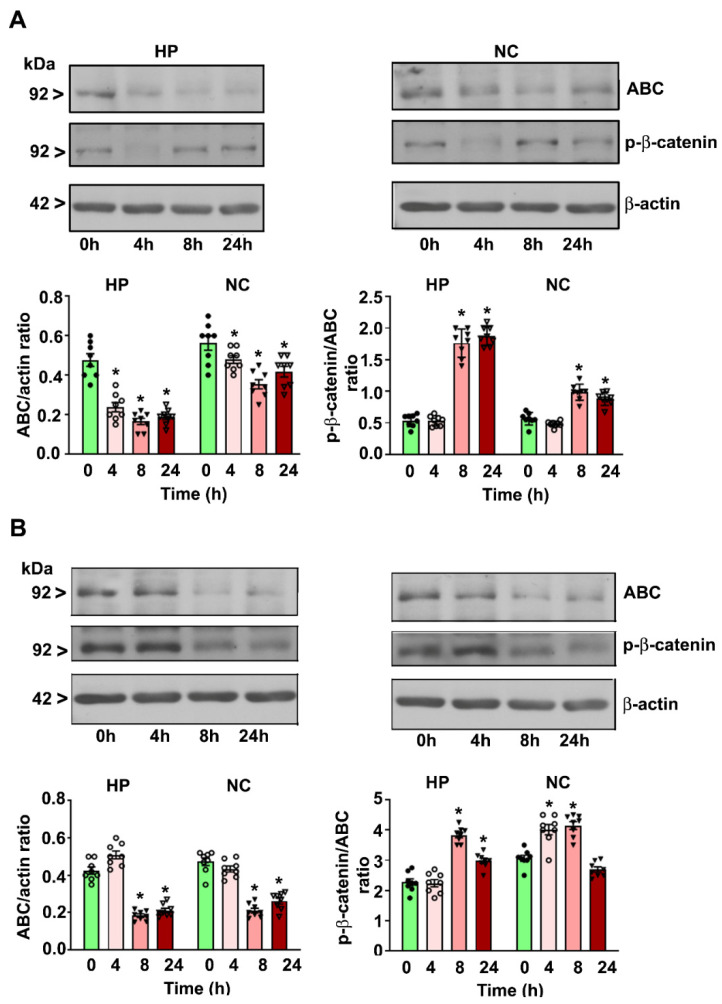
Decreased cytosolic active-β-catenin (ABC) and p-β-catenin protein levels in the P7 mouse HP and NC tissues in response to moderate- and high-dose ethanol exposure. The HP and NC cytosolic extracts obtained 4–24 h after the first saline or moderate-dose (**A**)/high-dose (**B**) ethanol exposure. The ABC and p-β-catenin protein levels were determined using Western blot analysis. The protein samples were equally loaded, confirmed with Ponceau S staining, and normalized to β-actin. For the 0 h ethanol group, saline was administered. Error bars, SEM (* *p* < 0.05 vs. the saline [0 h] group, *n* = 8 pups/group).

Both moderate- (Figure 2A) and high-dose (Figure 2B) ethanol reduced the ABC protein levels in a time-dependent manner in the HP (moderate-dose: F_3, 28_ = 26; high-dose: F_3, 28_ = 32, *p* < 0.05) and NC (moderate-dose; F_3, 28_ = 29, *p* < 0.05; high-dose: F_3, 28_ = 21, *p* < 0.05) at the 4–24 h (after the first ethanol administration) time points compared to the levels in the saline group (0 h) (one-way ANOVA with Bonferroni’s post hoc test).

### 4.2. P7 Ethanol Exposure Increases the Cytosolic p-β-catenin/ABC Ratios in the HP and NC

Moderate-dose ethanol enhanced the p-β-catenin/ABC protein ratios in the HP (F_3, 28_ = 26, *p* < 0.05) and NC (F_3, 28_ = 29, *p* < 0.05) at 8 and 24 h (Figure 2A) (after the first ethanol administration) compared to the levels in the saline group (0 h) (one-way ANOVA with Bonferroni’s post hoc test). High-dose ethanol increased the p-β-catenin/ABC protein ratios in the HP (F_3, 28_ = 20, *p* < 0.05) at 8 and 24 h and in the NC (F_3, 28_ = 22, *p* < 0.05) at 4 and 8 h but not at 24 h (Figure 2B) (after the first ethanol administration) compared to the levels in the saline group (0 h).

### 4.3. P7 Ethanol Exposure Reduces the Nuclear ABC Levels in the HP and NC

**Figure 3:**
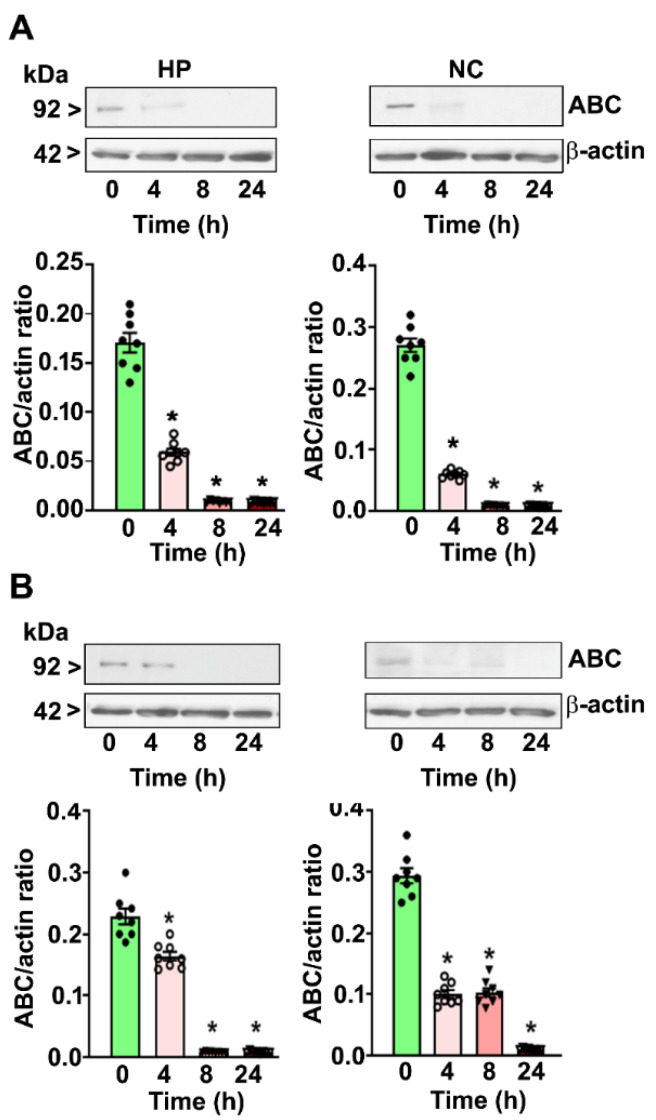
Reduced nuclear ABC protein levels in the P7 mouse HP and NC tissues in response to moderate- and high-dose ethanol exposure. The HP and NC nuclear extracts obtained 4–24 h after the first saline or moderate-dose (**A**)/high-dose (**B**) ethanol exposure. The ABC protein levels were analyzed using Western blot analysis. The protein samples were equally loaded, confirmed with Ponceau S staining, and normalized to β-actin. For the 0 h ethanol group, saline was administered. Error bars, SEM (* *p* < 0.05 vs. the saline [0 h] group, *n* = 8 pups/group).

Both moderate- (Figure 3A)and high-dose (Figure 3B) ethanol decreased the nuclear ABC protein levels in a time-dependent manner in the HP (moderate-dose: F_3, 28_ = 36; high-dose: F_3, 28_ = 38, *p* < 0.05) and NC (moderate-dose; F_3, 28_ = 24, *p* < 0.05; high-dose: F_3, 28_ = 26, *p* < 0.05) at the 4–24 h (after the first ethanol administration) time points compared to the levels in the saline group (0 h) (one-way ANOVA with Bonferroni’s post hoc test).

### 4.4. CB1R Blockade Mitigates the PEE-induced Loss of ABC Expression in the HP and NC

**Figure 4:**
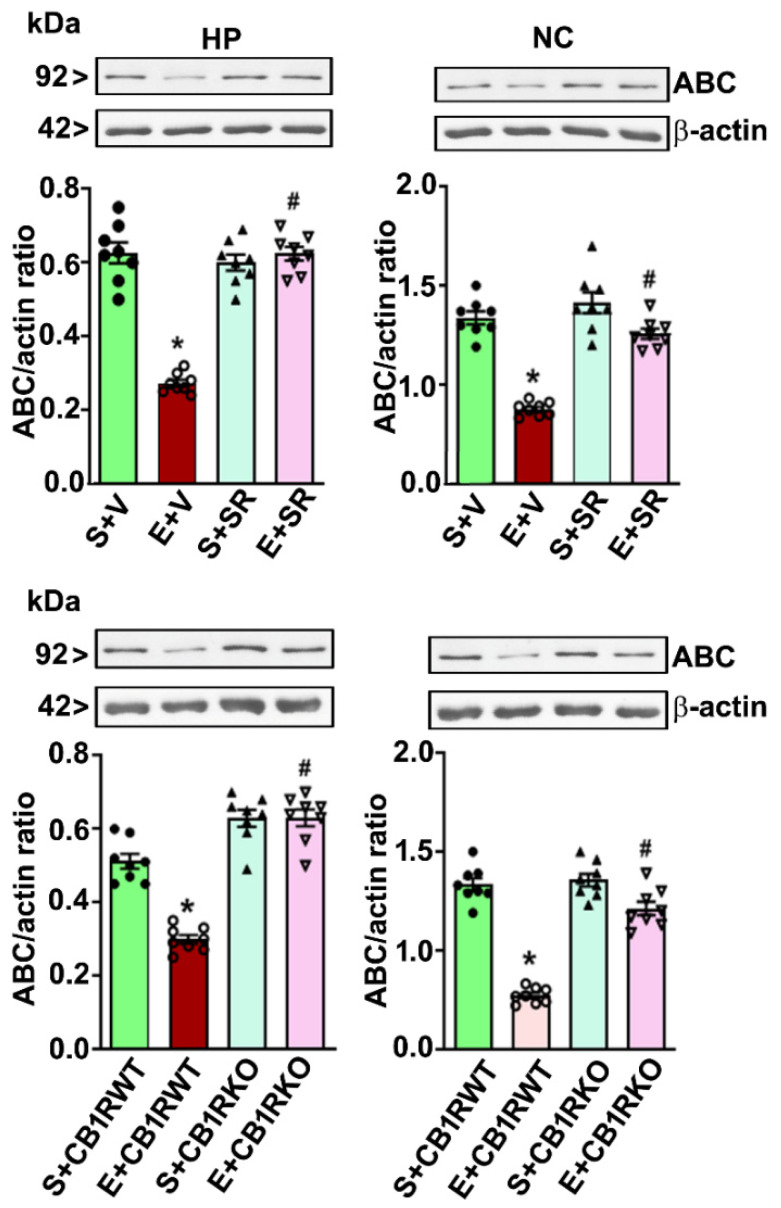
Preadministration of SR or genetic deletion of CB1Rs mitigates high-dose ethanol-induced loss of cytosolic ABC in the neonatal mouse brain. Western blot analysis of the ABC proteins in the HP and NC cytosolic extracts obtained 8 h after saline or ethanol treatment from different group (S + V, E + V, S + SR and E + SR; S + CB1RWT, E + CB1RWT, S + CB1RKO, and E + CB1RKO). The protein samples were equally loaded, confirmed with Ponceau S staining, and normalized to β-actin. Error bars, SEM (* *p* < 0.05 vs. S + V or S + CB1RWT group; # *p* < 0.05 vs. E + V or E + CB1RWT group, *n* = 8 pups/group).

**Figure 5:**
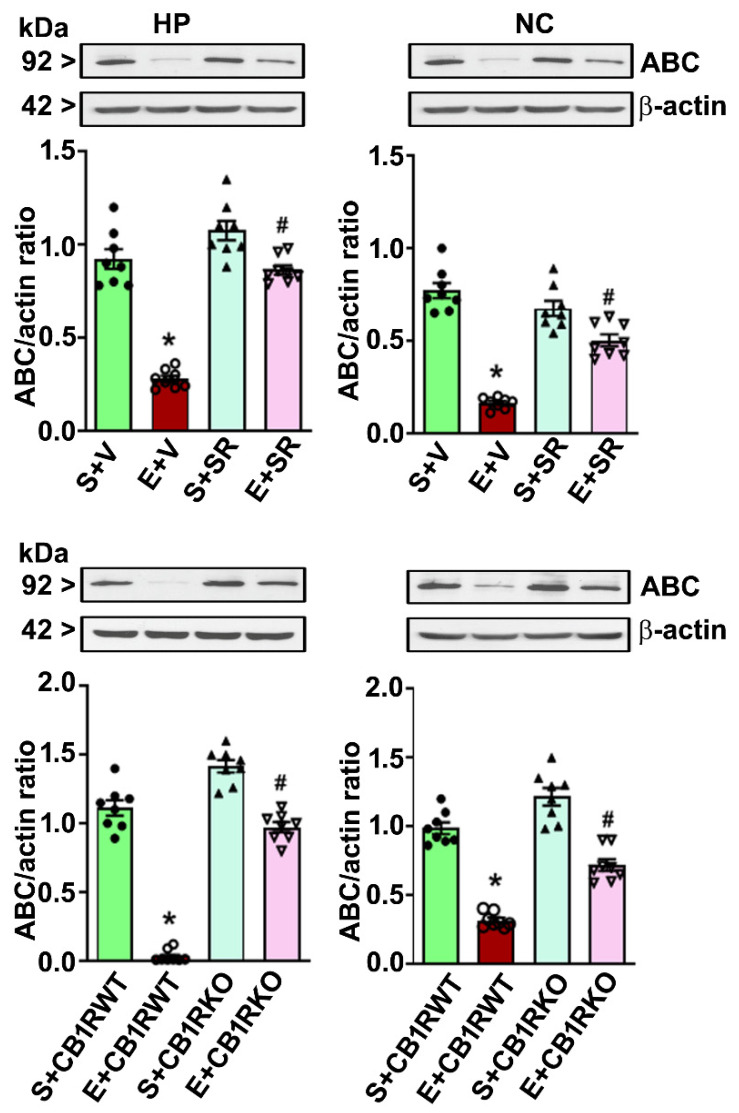
Pharmacological inhibition or genetic deletion of CB1Rs mitigates the loss of nuclear ABC caused by high-dose ethanol exposure in P7 mice. The ABC protein levels were evaluated by Western blot analysis in the nuclear fractions of the HP and NC samples from the different treatment groups (S + V, E + V, S + SR and E + SR; S + CB1RWT, E + CB1RWT, S + CB1RKO and E + CB1RKO). Error bars, SEM (* *p* < 0.05 vs. the S + V or CB1RWT + S group; # *p* < 0.05 vs. the E + V or CB1RWT + E group, *n* = 8 pups/group).

Because pharmacological blockade or genetic ablation of CB1R blocks PEE to induce neurodegeneration without affecting ethanol metabolism in P7 mice [19,20,25,32,54], we determined the role of CB1R in the PEE-induced loss of ABC protein expression in the cytosolic (Figure 4) as well as the nuclear fractions (Figure 5). Preadministration of SR significantly mitigated the high-dose PEE-induced loss of ABC protein expression (*p* < 0.05) in the HP (cytosolic: *F*_1,20_ = 19, *p* < 0.05; nuclear: *F*_1,20_ = 29, *p* < 0.05) and NC (cytosolic: *F*_1,20_ = 17, *p* < 0.05; nuclear: *F*_1,20_ = 39, *p* < 0.05). Additionally, high-dose PEE in the CB1R KO mice failed to induce the loss of ABC in the HP (cytosolic: *F*_1,20_ = 32, *p* < 0.05; nuclear: *F*_1,20_ = 36, *p* < 0.05) and NC (cytosolic: *F*_1,20_ = 22, *p* < 0.05; nuclear: *F*_1,20_ = 19, *p* < 0.05) (one-way ANOVA with Bonferroni’s *post hoc* tests).

## 5. Discussion

β-Catenin is involved in controlling many of the cellular functions of the developing CNS and has been shown to orchestrate neuronal differentiation, neuron death/survival, axonal elongation, synapse formation, and plasticity, neurotrophin transcription, neurogenesis, and regeneration [55,56,57,58,59]. A significant outcome of this investigation is the demonstration that CB1R regulates β-catenin protein levels in postnatal ethanol-induced neurodegeneration in the neonatal brain. The ability of postnatal ethanol to dysregulate β-catenin also represents a novel mechanism by which developmental ethanol affects neuronal death, survival, and neuronal maturation. Additionally, these findings highlight CB1R-mediated β-catenin as a mediator of developmental ethanol neurotoxicity within postnatal neurons. A previous study showed that acute ethanol exposure of chick embryos reduced β-catenin via a calcium/calmodulin-dependent protein kinase II (CaMKII)-mediated mechanism in early neural progenitors [60].

Numerous studies report alterations in β-catenin in response to ethanol insult; nonetheless, most of these studies used chronic ethanol exposure and, therefore, may manifest cellular adaptation in reaction to ethanol challenge. Chronic ethanol exposure significantly suppressed β-catenin signaling and the expression of Wnt effectors in bone [61,62,63]. In neurons, chronic ethanol exposure reduced the total β-catenin content in cultured hippocampal neurons [64] whereas elevating total β-catenin in the frontal cortex of chronic alcoholics [65]. These studies failed to differentiate between the cytosolic and nuclear fractions, and thus, the functional implications of those alterations are unclear. Acute ethanol exposure rapidly depleted nuclear β-catenin from osteoblasts [61] and loss of β-catenin/TCF signaling, followed by the loss of neural crest cells in chick [66] and murine models of fetal alcohol syndrome [67,68,69,70] similar to our findings in postnatal mouse HP and NC. Further, overexpression of β-catenin prevented the ethanol-induced neural crest apoptosis in chick embryos. On the other hand, overexpression of ΔTCF, which causes β-catenin loss-of-function also caused apoptosis [66]. Genetic conditional deletion of β-catenin also caused widespread cranial neural crest apoptosis, followed by brain malformation [71]. These findings together suggest that loss of β-catenin/TCF signaling may have a broader critical role in the action of acute ethanol exposure during different stages of brain development. Wnt/β-catenin signaling is vital for many cellular events, including cell growth and proliferation, cell fate, differentiation, and cellular adhesion in embryonic, fetal, and adult tissues. These findings suggest that ethanol-induced inhibition of canonical Wnt signaling may contribute to delayed maturation of the brain through adulthood. Therefore β-catenin signals are thus a novel, potential target of developmental ethanol exposure.

We and others showed earlier that PEE causes significant activation of caspase-3 in neonatal mice [19,20,54], and this neuroapoptosis-induced damage contributes to the impaired neuronal plasticity, learning, and memory [19,20,54] that resembles cognitive deficits in individuals who experienced alcohol exposure during early development [19,20,54]. Previous studies have separately shown that two upstream events that are necessary for this loss of β-catenin are the activation of CB1R and caspase-3, as preadministration of a pancaspase-3 inhibitor [54] or blockade of CB1R activity before PEE prevents caspase-3 activation [19,20]. Here, we showed that blockade of CB1R activity mitigates the loss of β-catenin. These observations suggest that the CB1R-mediated activation of caspase-3 is responsible for the loss of transcriptionally active β-catenin.

A previous study in non-neuronal cells showed that activation of CB1R by exogenous agonists increased the phosphorylation of β-catenin, followed by loss of cytosolic and nuclear β-catenin [34]. β-catenin functions to control the transcription of genes via the binding of a complex of β-catenin and the TCF family of transcription factors to particular promoter regions. The decrease in nuclear β-catenin by PEE and its mitigation by blocking CB1R activity suggested that β-catenin transcriptional activity might be under the control of the CB1R signaling pathway in neurodegenerating conditions. These findings suggest a novel mechanism by which neurons are sensitive to ethanol during the postnatal developmental period.

These findings raise the query as to how PEE abolished β-catenin activity. Protein degradation is the most studied mechanism for β-catenin regulation in the brain and other tissues. The β-catenin complex is phosphorylated by a destruction complex consisting of Axin, adenomatous polyposis coli, and glycogen synthase kinase-3, causing proteasomal degradation. Wnt activation causes this destruction complex to dissociate, leading to the stabilization of β-catenin [35,72]. Moreover, β-catenin degradation is regulated by other factors, such as protein kinase A and CaMKII [36,60,73]. Additionally, β-catenin has been shown to be proteolytically cleaved via caspase-3 during apoptosis [74]. β-catenin has been suggested to play a critical role in the regulation of apoptosis. It was shown that the overexpression of β-catenin deletion constructs with truncated N- and C-terminal regions led to enhanced apoptosis in rat hippocampal neurons [75]. The overexpression of a dominant-negative TCF also caused apoptosis, suggesting that the inhibition of β-catenin/TCF signaling promotes apoptosis. Many neurodegeneration-causing compounds also induce the loss of β-catenin in many cells, including neurons. Environmental Parkinson’s disease (PD) toxins and pesticides were shown to downregulate β-catenin signaling in rodents, non-human primates, and human PD [76,77]. The β-catenin protein levels were also found to be significantly reduced in AD patients carrying presenilin-1 (PS-1) inherited mutations [75] and in other types of AD-related neurodegeneration [39,41,78]. Therefore, proteolytic cleavage of β-catenin may not only be an effect of apoptosis but may also cause the apoptotic program.

Growing studies suggest that actin and actin-binding proteins impact gene expression at various levels [79,80]. For example, in association with RNA polymerases or other transcriptional effectors, it fosters the likelihood that β-catenin regulates cell-cell adhesion and gene expression via shared associations with actin. In the future, the evaluation of catenin interactions in the nucleus, both at the protein and the whole-genome expression levels, will provide new novel links that relate to processes such as cellular reprogramming, and the contributions of nuclear actin to these processes. In this respect, it is fascinating that several catenin nuclear partners have a strong functional association with the regulation of chromatin. For instance, *transcriptional* repressor element-1 (RE-1) silencing transcription factor (REST) and CoREST support several repressive histone-modifying activities [81,82,83] with p120 catenin (catenin partner) alleviating such repression at some gene promoters. Therefore, it appears that the larger biology of nuclear catenin’s role in chromatin regulation is of future interest.

In summary, the outcome of this study suggests that CB1R can regulate neurodegeneration during a sensitive period of brain development. The findings also indicated that PEE-induced neurodegeneration is mediated through the inactivation of β-catenin. Overall, our results revealed the participation of CB1R signaling in the postnatal ethanol-induced inactivation of β-catenin transcriptional activity in the developing brain. The crosstalk of the CB1R signaling and β-catenin transcriptional activity pathways suggests new potential strategies for therapy.
